# The associations among gratitude, job crafting, teacher-student relationships, and teacher psychological well-being

**DOI:** 10.3389/fpsyg.2024.1329782

**Published:** 2024-01-19

**Authors:** Xue Zheng, Hezi Huang, Quanda Yu

**Affiliations:** ^1^Public English Teaching and Research Department, Qiqihar University, Qiqihar, China; ^2^College of the Environment and Ecology, Xiamen University, Xiamen, China; ^3^Radiochemotherapy Department, First Hospital of Qiqihar, Qiqihar, China

**Keywords:** gratitude, job crafting, teacher-student relationships, teacher psychological well-being, Chinese EFL teachers

## Abstract

**Introduction:**

This study explores the complex dynamics among gratitude, job crafting, teacher psychological well-being, and teacher-student relationships within the context of Chinese English as a Foreign Language (EFL) teachers.

**Methods:**

A sample of 456 Chinese EFL teachers participated in this study. Valid scales were administered to collect data on gratitude, job crafting, teacher psychological well-being, and teacher-student relationships. Structural Equation Modeling (SEM) was employed to investigate these relationships.

**Results:**

The findings reveal significant connections between gratitude, job crafting, teacher psychological well-being, and teacher-student relationships. SEM analysis demonstrates that gratitude and job crafting have direct effects on teacher psychological well-being. Furthermore, teacher-student relationships were identified as a mediator in these relationships.

**Discussion:**

This study underscores the importance of gratitude and job crafting in enhancing the psychological well-being of EFL teachers. It highlights the mediating role of positive teacher-student relationships in the associations between gratitude, job crafting, and teacher psychological well-being. These results have implications for the development of interventions and practices aimed at promoting gratitude, job crafting, and positive teacher-student relationships in the EFL teaching context.

## 1 Introduction

The transition from theoretical knowledge to the practical facets of teaching poses a significant challenge for novice educators (Day and Gu, 2007; Aldrup et al., 2017; Schmidt et al., 2017). This shift often leads to reduced levels of well-being among educators (Fernet et al., 2012; Wolomasi et al., 2019), impacting not just the quality of the teaching workforce but also profoundly affecting students’ academic progress (Carver-Thomas and Darling-Hammond, 2019; Li and Yao, 2022). The imperative to address teacher well-being becomes critical considering its multifaceted impact across the educational landscape.

Recent research has illuminated the pivotal role of gratitude in individuals’ psychological health, unveiling its negative associations with anxiety and depressive disorders (Chen et al., 2012; Lin, 2015; Sun et al., 2020). Conversely, gratitude shows positive correlations with various beneficial social and psychological adaptations, including elevated life quality, fulfillment, and a sense of belonging (Sun and Kong, 2013; Tian et al., 2015; Portocarrero et al., 2020; Huber et al., 2021). Characterized by a consistent focus on life’s affirmative aspects, dispositional gratitude emerges as a critical psychological trait intertwined with overall well-being (Wood et al., 2008a; Rash et al., 2011). Gratitude is widely acknowledged to be significant for interpersonal relationships and overall wellbeing, but it also has a special effect on how one feels about one’s job (Lanham et al., 2012; Young and Hutchinson, 2012; Di Fabio et al., 2017; Cain et al., 2019). While gratitude’s significance for interpersonal relationships and overall well-being is acknowledged, its specific impact on educators’ well-being within the teaching context remains an underexplored domain.

Concurrently, the realm of job design research, particularly regarding job crafting, has experienced a surge (Oldham and Hackman, 2010; Irfan et al., 2023; Sameer and Priyadarshi, 2023). Job crafting involves intentional alterations in job roles to align with individual preferences, fostering enhanced job-related well-being and productivity (Bruning and Campion, 2019; Zhang and Parker, 2019; Lazazzara et al., 2020; Tims et al., 2022). While the potential benefits of job crafting are evident across various work settings, its implications for educators’ well-being within the teaching profession necessitate deeper exploration.

Teachers play a pivotal role in fostering student academic engagement through classroom interactions (Pianta et al., 2012; Quin, 2017; Nguyen et al., 2018; Longobardi et al., 2021). The quality of teacher-student relationships profoundly influences the learning environment, spurring active student participation and supporting academic advancement (Meng, 2021; Zhou, 2021). Despite substantial research on gratitude and job crafting, their direct interconnections and potential mediation through teacher-student relationships within the teaching context remain relatively unexplored domains.

In this study, we aim to unravel the intricate connections between gratitude, job crafting, teacher-student relationships, and teacher psychological well-being. The theoretical underpinning of this investigation revolves around exploring how gratitude, as a positive psychological trait, interacts with job crafting behaviors among educators, subsequently influencing the quality of teacher-student relationships. We hypothesize that teachers, by exercising job crafting behaviors, can potentially modify their roles and responsibilities, impacting their interactions with students. We propose that teacher-student relationships act as a mediator, linking gratitude and job crafting to teacher psychological well-being. This study seeks to delve into the nuanced relationships among these variables, thereby shedding light on how these constructs intertwine within the unique professional landscape of Chinese English as a Foreign Language (EFL) teaching. Understanding these interconnections holds the promise of not only enriching the empirical knowledge but also offering valuable insights into strategies for fostering supportive teaching environments and enhancing both educator well-being and student outcomes.

## 2 The review of literature

### 2.1 Theoretical framework

The present study draws upon the Job Demands-Resources (JD-R) Model formulated by Bakker and Demerouti (2007) to illuminate the complex interconnections between gratitude, job crafting, teacher psychological well-being, and teacher-student relationships among Chinese EFL educators. This established theoretical framework has gained prominence for its comprehensive approach in understanding the dynamics influencing employee well-being within the workplace context (Schaufeli, 2017).

The JD-R Model presents a comprehensive framework categorizing workplace factors into two distinct but interrelated dimensions: job demands and job resources. Job demands constitute elements within the work environment that necessitate physical, cognitive, or emotional effort from employees and are often linked to stress, exhaustion, and potential burnout (Bakker et al., 2001; Bakker and Demerouti, 2017). Conversely, job resources encompass factors that facilitate the achievement of work-related goals, buffer the negative impact of job demands, and foster work engagement and satisfaction (Bakker and Demerouti, 2007).

Gratitude, regarded as a positive psychological trait, aligns with the concept of personal resources within the JD-R Model. It is anticipated to function as a coping mechanism, potentially mitigating the detrimental effects of job demands on teachers’ psychological well-being (Chan, 2010a; Cain et al., 2019). Previous studies have underscored gratitude’s role as a protective factor against stress, burnout, and psychological distress (Wood et al., 2008; Emmons and Mishra, 2011). In the context of the JD-R Model, gratitude is hypothesized to contribute significantly to teachers’ psychological well-being by counteracting the negative impacts of job demands.

Furthermore, job crafting, conceptualized as an adaptive strategy within the JD-R Model, empowers individuals to proactively modify their work environments, aligning them more closely with their needs, preferences, and strengths (Alonso et al., 2019; Dreer, 2022). It serves as a motivational resource enhancing work engagement and well-being by allowing individuals to alter job demands and resources to suit their professional preferences (Tims and Bakker, 2010). In this study, job crafting is seen as a mechanism through which teachers actively modify their job roles and responsibilities, potentially influencing their psychological well-being by adjusting the demands and resources associated with their work (Bruning and Campion, 2019).

Teacher-student relationships, fundamental in the teaching profession, are viewed as both job demands and job resources within the JD-R Model. Positive relationships can serve as resources, fostering engagement and support, while challenging interactions might represent demands leading to stress and emotional exhaustion (Bakker et al., 2005; Bakker and Demerouti, 2007; Farhah et al., 2021; Zheng, 2022). This study aims to explore how teacher-student relationships operate as both demands and resources, mediating the associations between gratitude, job crafting, and teacher psychological well-being among EFL teachers. These relationships play a pivotal role in shaping the teaching environment, influencing not only teacher well-being but also potentially impacting student outcomes and the overall classroom atmosphere.

By adopting the JD-R Model as the theoretical framework, this study endeavors to offer a comprehensive understanding of the intricate mechanisms linking gratitude, job crafting, teacher-student relationships, and teacher psychological well-being among Chinese EFL teachers. This theoretical lens provides a structured approach to comprehend the complex interactions between various elements within the teaching context, paving the way for a nuanced exploration of factors influencing teacher well-being and classroom dynamics.

### 2.2 Wellbeing

According to Compton et al. (1996), well-being is a multifaceted concept pertaining to optimal experience and functioning. Within psychology, there are two distinct ways to define psychological well-being: the initial philosophical class, known as the subjective well-being approach or hedonism, defines well-being as fulfillment or enjoyment (Kahneman et al., 1999; Deci and Ryan, 2008). Three factors make up this subjective well-being: life satisfaction, the existence of a pleasant mood, and the absence of a negative mood. These three factors are typically taken into account together and referred to as “happiness” (Ryan and Deci, 2001). Empirical psychology has historically concentrated on subjective well-being because of its correlation with both physical and mental health (Senol-Durak and Durak, 2011; Donoso et al., 2021). Studies have shown that a range of psychological illnesses are associated with greater levels of life satisfaction, a sense of optimism, faith, and happiness (Seligman et al., 2006), and this method places equal emphasis on the choices and joys of the intellect and body. The eudaimonic tradition, on the other hand, offers a different view of psychological well-being and contends that it is primarily about realizing one’s own abilities (Ruini and Ryff, 2016). Eudaimonism, or psychological well-being, refers to the degree to which people believe they are in authority over their lives, that their behavior is meaningful and valuable, and that they have beneficial relationships with other people. This term conveys the idea that recognizing or meeting one’s true nature, or daimon, is the ultimate goal of well-being (Annamalai et al., 2020; Nikbin et al., 2021).

Psychological well-being, a critical facet of overall well-being, encompasses an individual’s emotional, cognitive, and psychological state, reflecting their subjective appraisal of life satisfaction and fulfillment (Diener et al., 2010; Nikbin et al., 2021). For educators, psychological well-being holds particular significance, as it directly impacts their ability to effectively navigate the challenges inherent in the teaching profession (Yildirim, 2015; Zhang et al., 2023). In the context of teaching, psychological well-being encompasses various dimensions. A key dimension is job satisfaction, reflecting a teacher’s contentment with their professional role and the fulfillment derived from their work (Skaalvik and Skaalvik, 2017; Derakhshan et al., 2022; Zheng et al., 2022).

Also, instructors’ job satisfaction and exhaustion are significantly influenced by their emotional instruction (Yildirim, 2015; Zhang et al., 2023). Emotions are the precursors of well-being, according to the broaden-and-build hypothesis of pleasant emotions (Tugade et al., 2004). Pleasurable feelings, like well-being, increase cognitive and attention spans as well as social and psychological capital that contributes to a person’s overall wellbeing by lowering burnout and raising job satisfaction among educators (Huang et al., 2019; Zheng et al., 2022; Fathi et al., 2023b). Negative emotions, such as anger and anxiety, on the other hand, cause people to become less focused and less able to develop multiple psychological capabilities, such as resilience. This can lead to a decrease in wellbeing, such as in teachers who are more likely to experience burnout and be dissatisfied with their work (Chen, 2019; Greenier et al., 2021; Nalipay et al., 2022; Xiyun et al., 2022).

Recognizing the significance of psychological well-being, this study seeks to explore the interplay between gratitude, job crafting, and psychological well-being among EFL educators. By understanding how these factors interact and influence psychological well-being, this research aims to provide valuable insights for educators, educational institutions, and policymakers seeking to enhance the overall well-being of teachers and, consequently, the quality of education provided to students.

### 2.3 Job crafting

Job crafting, a concept rooted in the broader field of organizational psychology, is a proactive and self-initiated approach through which employees redesign and customize their job roles to better align with their personal strengths, preferences, and goals (Wrzesniewski and Dutton, 2001; Zhang and Parker, 2019). This process empowers individuals to exert greater control over their work environment, ultimately shaping their occupational experiences and enhancing their overall well-being (Tims et al., 2015). In the context of teaching, job crafting offers educators the opportunity to tailor their roles and responsibilities to optimize their teaching practices and, subsequently, their psychological well-being (Qi et al., 2016).

One of the fundamental aspects of job crafting is the alteration of job tasks. Teachers can modify their instructional approaches, curriculum design, and classroom management techniques to better suit their unique teaching style (Tims et al., 2012; Peral and Geldenhuys, 2016). By doing so, educators can create a teaching environment that aligns with their strengths and preferences, thus fostering a sense of autonomy and competence.

Additionally, teachers can engage in relational job crafting, which involves building and enhancing relationships with students, colleagues, and school administration (Tims et al., 2015; Dreer, 2022). For example, educators can initiate collaborative projects, seek out professional development opportunities, or engage in mentoring relationships. These interpersonal connections can contribute to job satisfaction, emotional well-being, and a sense of support and belonging within the school community (Alonso et al., 2019).

The Job Demands-Resources (JD-R) model provides a theoretical foundation for understanding the implications of job crafting for teacher well-being. According to this model, job resources, such as autonomy and social support, can act as buffers against job demands, ultimately fostering well-being and engagement (Bakker and Demerouti, 2007; Liu et al., 2023). In the teaching profession, job crafting represents a resource by allowing teachers to modify their roles to align with their preferences and strengths.

Job crafting was divided into three categories by Wrzesniewski and Dutton (2001) based on whether it involved altering the task’s scope, relationships, or significance of the work, respectively. By broadening the conceptualization of Wrzesniewski and Dutton (2001) from a JD-R perspective (Bakker et al., 2001; Xanthopoulou et al., 2007), workers can use task, relational, and cognitive crafting to match job demands and resources with their own capabilities and needs. These strategies involve raising job resources along with difficult demands or lowering hindrance demands. Task crafting essentially entails adjusting the quantity, nature, and kind of employment activities as well as the way time and energy are distributed across them (Tims et al., 2012; Bruning and Campion, 2019; Hu et al., 2020). According to Slemp and Vella-Brodrick (2013), relational crafting entails modifying the quantity or quality of social encounters. As stated by Wrzesniewski and Dutton (2001), cognitive crafting is manipulating one’s perspective on or thought process on labor. The JD-R paradigm suggests that job crafting can improve employees’ person-job fit by promoting an equilibrium among job demands and job resources. This, in turn, can lead to improved job satisfaction and favorable organizational results (Tims et al., 2015; Harju et al., 2021).

Prior research has indicated a favorable correlation among job crafting and a number of results for workers, including job performance, person-job fit, involvement in the workplace, and fulfillment with work. According to Leana et al. (2009), educators who craft their jobs have a greater chance of receiving excellent marks for their level of care from students. Kim et al. (2018) discovered a positive correlation between job satisfaction and the job crafting done by employees. Task, relational, and cognitive crafting were all examined by Slemp and Vella-Brodrick (2013), who proposed that all three dimensions—aside from the task facet in the latter study—were related to job satisfaction. Job crafting has been found by Slemp and Vella-Brodrick (2014) to be positively correlated with the satisfaction of intrinsic needs, which in turn improves employee well-being. Job crafting lessens stress and burnout since it entails adjusting the quantity and kind of work, the frequency and intensity of interpersonal contacts, and the meaning of their occupations to suit the choices and requirements of the employees (Bakker et al., 2004; Lesener et al., 2019).

In the context of job crafting and its influence on employee welfare, recent investigations have illuminated the correlation between these variables. Dreer (2022) delved into the realm of teacher well-being, probing the influential components of school climate and job crafting. Building upon this, Alonso et al. (2019) delved into an exploration of both individual and collaborative job crafting and their repercussions on the well-being of teachers in Spain. Van Wingerden et al. (2017) executed a study implementing an intervention centered on job crafting to foster employee well-being. Within the South African context, Peral and Geldenhuys (2016) scrutinized the ramifications of job crafting on the subjective well-being of high school teachers. Lastly, Wang et al. (2022) embarked on an investigation into the role of organizational backing and job crafting in augmenting the well-being of new math teachers, with a specific emphasis on the mediating influence of fundamental psychological needs.

These research endeavors collectively underscore the importance of job crafting as a contributory element to employee well-being, notably within the educational sector. Additionally, they bring to the forefront an array of facets and settings, including school climate, collaborative efforts, and organizational support, all of which can wield an impact on the interplay between job crafting and well-being. These observations highlight the intricate nature of job crafting and its implications for the comprehensive well-being of educators and workers within diverse contexts.

The study at hand explores the interplay between gratitude, job crafting, teacher-student relationships, and psychological well-being among EFL educators. By investigating the intricate relationships among these factors, we aim to provide empirical evidence of the profound impact of job crafting on teacher well-being and the quality of teacher-student relationships. The results of this study hold valuable implications for educators, educational institutions, and policymakers committed to enhancing teacher well-being and improving the educational experiences of students.

### 2.4 Gratitude

Gratitude, as a complex and multifaceted emotional state, has received increasing attention in the fields of positive psychology, well-being, and education (Gulliford et al., 2013; Garg, 2022). It involves the recognition and appreciation of positive aspects in one’s life and experiences (Wood et al., 2010). In the realm of teaching, cultivating gratitude can be a transformative force, impacting not only the educators themselves but also the students and the overall classroom environment (Chan, 2010b).

Gratitude, often characterized by feelings of thankfulness and appreciation, plays a significant role in teacher well-being (Chan, 2013). Research has shown that gratitude is closely linked to various dimensions of well-being, including life satisfaction, happiness, and overall psychological well-being (Emmons and McCullough, 2003; Portocarrero et al., 2020). For educators, these positive emotions associated with gratitude can contribute to job satisfaction, reduced stress, and overall psychological well-being (Komase et al., 2021).

Upon receiving assistance from someone else and considering that assistance to be costly, valuable, and selfless, one experiences gratitude (Bartlett and DeSteno, 2006; Wood et al., 2008b). Thus, showing gratitude frequently entails expressing appreciation for the helpful deeds of others (Tsang, 2007; Solom et al., 2017). According to the broaden-and-build theory of gratitude, people who are grateful may find that their repertoire of quickly generated thoughts and acts grows (Fredrickson, 2004; Gulliford et al., 2013). Moreover, gratitude may improve and expand one’s social and intellectual capacities, according to the broaden-and-build theory (Fredrickson, 2004; Zhen et al., 2021). Gratitude can reduce negative feelings even in an academic setting and is strongly correlated with positive assessments of oneself and life (Rash et al., 2011; Nezlek et al., 2017; Simons et al., 2020).

As a psychological characteristic, dispositional gratitude has been shown to support people in using adaptable and constructive coping mechanisms at work and is a strong predictor of job satisfaction (Bartlett et al., 2012; Tsang and Martin, 2019). Higher gratitude levels allowed people to devote more of their stored resources and capacity for pleasant emotions to their duties (Chen et al., 2021; Garg et al., 2022). Higher gratitude levels have been linked to increased creativity and organizational citizenship habits, as well as increased responsibility, enthusiasm, and an intense sense of obligations (Yang and Li, 2018). According to a meta-analysis study, those who have gratitude typically behave in a more proactive manner (He et al., 2018). According to Hu et al. (2011), job crafting is a form of proactive behavior that deviates from conventional top-down organizational structure and administrative assignment behavior. It is the action of staff members to proactively enhance their job procedures and materials (Niessen et al., 2016; Bruning and Campion, 2019).

By taking the initiative to create work content, alter work procedures, and enhance work quality in accordance with needs, employees engage in job crafting, which is a good work behavior. Employees that exhibit these constructive and proactive actions may find that their job has meaning and that their own values, motivation, and interests align with it (Zhao and Li, 2019; Chen et al., 2021). It has also been shown that job crafting raises staff members’ job satisfaction levels (Tims et al., 2015; Qi et al., 2016; Sakuraya et al., 2020).

An inclusive synthesis of recent research investigating the correlation between gratitude and well-being reveals a substantial association. In one instance, Chan’s (2010a) study scrutinized this connection within the milieu of Chinese educators in Hong Kong, concluding that expressions of gratitude and interventions centered around gratitude exerted a beneficial influence on their individual well-being. Subsequently, Chan (2013) expanded upon this subject by examining the subjective well-being of teachers in Hong Kong, underlining the substantial roles played by gratitude, forgiveness, and orientations toward happiness.

Shifting focus to a distinct context, Howells (2014) delved into the significance of gratitude in the realm of teacher-student relationships, elucidating its pivotal role in fortifying these interpersonal bonds. In a related vein, Komase et al. (2021) conducted a comprehensive systematic review, exploring the ramifications of gratitude interventions on the mental health and well-being of professionals. Their findings pointed to the favorable impacts of such interventions.

Furthermore, Chan’s (2010a) revisit of the concept of teacher burnout introduced a novel perspective, proposing the integration of positive interventions founded on gratitude and forgiveness. This forward-thinking approach suggested promising prospects in terms of alleviating burnout and enhancing the overall well-being of educators.

A number of investigations carried out in India has explored the multifaceted dimensions and impacts of appreciation across diverse spheres. Bansal et al. (2023a) scrutinized the Cyberbullying Attitude Scale (CBAS) among college students, evaluating its metric properties and its association with teasing and appreciation. Their subsequent inquiry (Bansal et al., 2023b) delved into the correlation between teasing based on body weight, symptoms of depression, and appreciation among university students, examining how appreciation might moderate the effects of teasing on mental well-being. Expanding on the theme of gratitude, Garg (2022) analyzed the interplay between appreciation and the enrichment of work-life balance among female employees, highlighting the intervening influence of psychological capital. Another study by Garg (2023) concentrated on validating the Transpersonal Gratitude Scale (TGS) and exploring the link between transpersonal appreciation, spiritual wellness, and distress. Also, the investigation led by Garg et al. (2022) probed the association between gratitude and vigor among students, investigating the mediating role of resilience. Additionally, Garg et al. (2023) explored the repercussions of toxic workplace environments on employees’ intentions to leave their jobs, examining how appreciation might potentially moderate this relationship.

Moreover, Garg and collaborators validated the Existential Gratitude Scale (EGS) in India, studying its connection with spiritual well-being and distress (Garg et al., 2023a). They also explored the relationship between appreciation, technostress, and adopting a positive perspective among the students (Garg et al., 2023b). Furthermore, Mahipalan and Garg (2023) examined how toxic workplace environments affect employees’ psychological capital, focusing on the potential moderating role of appreciation. Meanwhile, Mahipalan and Sheena (2019) investigated the spirituality of the workplace and subjective happiness among high school educators, highlighting the moderating function of appreciation. Lastly, Sarkar et al. (2023) explored whether gratitude can counteract toxic workplace settings, examining its plausible mediating role in enhancing psychological capital and ameliorating unfavorable workplace circumstances.

This study seeks to delve deeper into the role of gratitude in the well-being of EFL educators. By exploring the interplay between gratitude, job crafting, teacher-student relationships, and psychological well-being, we aim to provide empirical evidence of the profound impact of gratitude in educational contexts. Understanding the mechanisms through which gratitude influences teacher well-being and the quality of teacher-student relationships holds significant implications for educators, educational institutions, and policymakers committed to enhancing the well-being of teachers and the educational experiences of students.

### 2.5 Teacher-student relationship

Teacher-student relationships represent a critical element within the educational landscape, recognized for their profound influence on both students and teachers (Roorda et al., 2011; Aldrup et al., 2018; Longobardi et al., 2021). The quality of these relationships encompasses the emotional and interpersonal bonds between teachers and their students, playing a pivotal role in shaping the teaching and learning processes (Fabris et al., 2023). Relationships between teachers and students are frequently cited as one of the main motivators for continuing in the field (Kelchtermans and Deketelaere, 2016; Longobardi et al., 2022). Connections with learners were the primary source of inspiration and enjoyment for instructors, according to Hargreaves (2000) in-depth interviews with 60 of them. The results showed that instructors in elementary schools had more emotionally intense relationships than those in high schools; however, this was true for both primary and secondary school instructors. Elementary school instructors reported experiencing higher levels of both positive and negative emotions, as well as more instances of annoyance and dissatisfaction. Secondary educators frequently spoke about their interpersonal interactions with students in terms of recognition and deference. It may be a little more challenging for secondary school instructors to have a personal connection with their students because of the structured framework of secondary school. As a result, secondary instructors reported feeling more alienated from their students and more frequently misunderstood by them, which was frequently cited as a cause of unfavorable feelings. These interviews demonstrated the importance educators place on developing personal connections with the students in their classes, and showed how hostile or contentious interactions can jeopardize educators’ personal and professional well-being.

Descriptive and correlational studies have additionally provided some evidence supporting the significance of positive teacher-student connections for instructors’ well-being (Lin et al., 2022). Shann (1998) examined teacher satisfaction using data from a three-year study on the efficiency of schools in four sizable urban middle schools. Instructors ranked teacher-student interactions as the most significant of 14 critical criteria, including educational programs, job stability, instructor autonomy, acknowledgment of teachers’ accomplishments, and relationships at work, according to data from both questionnaires and interviews. Positive teacher-student connections were also found to be the most fulfilling. This aligns with educators’ strong sense of personal obligation to their students. According to correlational studies, there is some evidence to support the notion that teacher wellbeing is related to teacher perceptions of conflict and intimacy in relationships with specific students. Research has shown the instructors’ experiences with conflict, but not closeness, are somewhat correlated with their efficacy beliefs (Wang et al., 2017). When conflict exceeds expectations due to instructors perceptions of problematic behavior in students, teachers’ self-reported depression is also somewhat correlated with these findings (Buyse et al., 2008; Jerome et al., 2009).

Moreover, constructive teacher-student relationships contribute to dynamic instructional methods by acting as an external source of motivated change (Sun and Shi, 2022; Xie and Guo, 2023). As some may have experienced, a teacher’s performance and the students’ cooperative actions can have an important influence on a student’s motivation and success in a course (Den Brok et al., 2004). Theorists of social motivation contend that students who receive social support from teachers will form strong motivating beliefs that will promote active learning, dedication, and excellent performance (King, 2015; Longobardi et al., 2022). According to Lapointe et al. (2005), a consistent rapport between teachers and students can effectively mitigate the consistently observed decline in students’ motivation to take charge of their own learning over time. Interpersonal psychology, which views an individual’s behavior in terms of conditioned causality and mutual impacts, is based on the assumption that the foundation of instructor-learner relationships plays an important role in improving inspiration and extending learning returns (Jackson-Dwyer, 2013; Meng, 2021).

A comprehensive review of recent studies examining the connection between the teacher-student relationship and its effects on teacher well-being uncovers a complex interplay between these factors. Aldrup et al. (2018) conducted an investigation into the impact of student misbehavior on teacher well-being and found that the quality of the teacher-student relationship serves as a significant mediating factor. Their results indicate that the teacher-student relationship plays a mediating role in the link between student misbehavior and teacher well-being.

In situations marked by crises, Falk et al. (2022) delved into the dynamics of teacher-student relationships and teacher well-being. They underscored the concept that teachers frequently take on their students’ issues as their own, shedding light on the intricate relationship between teacher-student connections and teacher well-being in demanding circumstances. Additionally, Farhah et al. (2021) examined the influence of teaching experience as a moderator in the association between student-teacher relationships and teacher subjective well-being. Their study highlighted the importance of teaching experience in shaping how the teacher-student relationship affects teacher well-being, recognizing the nuanced nature of this association. Zheng (2022) concentrated on the promotion of students’ well-being and emphasized the mediating role of teacher interpersonal behavior and teacher-student relationships. Their research illuminated the pivotal role played by teacher-student relationships and teachers’ behavior in fostering students’ well-being, subsequently indirectly impacting teacher well-being.

To sum up, these collective inquiries underscore the intricate and multifaceted connection between teacher-student bonds and teacher well-being. They demonstrate that the quality of teacher-student relationships, student misbehavior, teaching experience, and teacher interpersonal behavior all have essential roles in influencing teacher well-being. These findings stress the significance of nurturing positive teacher-student relationships and the necessity for support systems in demanding educational settings to enhance teacher well-being.

### 2.6 The present study

In this research, we undertake an empirical investigation to rigorously examine and evaluate the hypotheses that follow. Our aim is to contribute to the understanding of how gratitude, job crafting, and teacher-student relationships collectively influence teacher psychological well-being. By probing these relationships, we seek to shed light on the intricate dynamics within the EFL education, providing insights that can inform interventions and practices to enhance the well-being of EFL teachers.

*H1:* Job crafting is directly related to EFL teacher well-being.

Job crafting, regarded as a proactive approach, empowers employees, including teachers, to dynamically modify their job roles and responsibilities in alignment with their preferences and strengths (Van Wingerden et al., 2017; Dreer, 2022). Through job crafting, teachers gain the agency to customize their teaching practices, pursue professional development avenues, and initiate collaborative projects, fostering a sense of autonomy and control over their professional roles (Alonso et al., 2019; Wang et al., 2022). This enhanced sense of autonomy and control has consistently demonstrated a positive association with elevated job satisfaction and an overall improved sense of well-being among employees (Van Wingerden et al., 2017; Alonso et al., 2019). Furthermore, research specifically focused on job crafting interventions, such as the study by Van Wingerden et al. (2017), underscores the effectiveness of such interventions in enhancing employee well-being, highlighting the direct relevance and applicability of job crafting concepts to the context of teacher well-being.

*H2*: Gratitude is directly associated with EFL teacher well-being.

Gratitude interventions and practices have consistently demonstrated a robust positive correlation with heightened levels of life satisfaction, increased happiness, and an overall enhanced sense of well-being among individuals (Chan, 2013). In the specific context of teaching, where educators confront multifaceted challenges and stressors, the cultivation of gratitude emerges as a valuable resource (Chan, 2010a, Howells, 2014; Komase et al., 2021). Encouraging teachers to recognize and appreciate the positive aspects inherent in their profession, such as the profound impact on students’ lives, serves as a pivotal strategy to redirect attention from the inevitable stressors and complexities of teaching (Chan, 2010b). This shift in perspective significantly contributes to elevated job satisfaction, mitigated burnout, and ultimately fosters an enriched sense of well-being (Howells, 2014; Komase et al., 2021). Extensive literature substantiates the notion that gratitude serves as a potent positive emotion capable of significantly enhancing overall well-being, underscoring its particular relevance and significance within the realm of teacher well-being.

*H3*: Teacher-student relationship mediates the relationship between job crafting and EFL teacher well-being.

Substantial evidence supports the concept of teacher-student relationships as a mediator between job crafting and teacher well-being. Aldrup et al. (2018) demonstrated that the quality of teacher-student relationships can mediate the impact of student misbehavior on teacher well-being, emphasizing the significant role these relationships play in shaping teachers’ psychological outcomes. Job crafting, by providing teachers with autonomy and control over their tasks and responsibilities, empowers them to foster more positive and supportive interactions with their students (Wang et al., 2022). The ability to customize their roles and duties can positively influence the quality of teacher-student relationships, enriching the emotional connection and interpersonal dynamics (Aldrup et al., 2018; Farhah et al., 2021; Falk et al., 2022; Zheng, 2022).

As evidenced, positive teacher-student relationships are linked to lower stress levels, improved job satisfaction, and overall enhanced well-being among educators (Aldrup et al., 2018; Zheng, 2022). Therefore, it is reasonable to infer that the impact of job crafting on teacher well-being may be influenced by the quality of teacher-student relationships. This hypothesis suggests that the effect of job crafting on teacher well-being is channeled through the intermediary role of teacher-student relationships, implying that these relationships could significantly shape the impact of job crafting on teacher well-being.

*H4:* Teacher-student relationship mediates the relationship between gratitude and EFL teacher well-being.

The hypothesis asserting that the teacher-student relationship mediates the link between gratitude and teacher well-being finds strong support within existing literature. Gratitude tends to foster a positive and empathetic attitude in teachers, significantly impacting their interactions with students (Chan, 2010a; Cain et al., 2019). A robust teacher-student relationship characterized by trust and positive engagement holds a direct correlation with teacher well-being (Howells, 2014). When educators experience a sense of connection and positive engagement with their students, it significantly contributes to their overall well-being and job satisfaction (Aldrup et al., 2018; Zheng, 2022; Fathi et al., 2023b).

Gratitude acts as a catalyst in nurturing these valuable connections, aligning with the principles of the J-DR model (Bakker et al., 2001; Bakker and Demerouti, 2007) by functioning as a resource that elevates the quality of teacher-student relationships. From this perspective, the mediated relationship between gratitude and teacher well-being emphasizes the critical role of cultivating positive teacher-student relationships in enhancing teachers’ psychological well-being and job satisfaction.

## 3 Methods

### 3.1 Participants and procedures

Utilizing a convenient sampling approach, 456 English teachers hailing from diverse regions across different provinces in China were extended invitations to partake in this study. The participants constituted a diverse range of educational settings, encompassing public and private schools, language academies, and international schools, thus guaranteeing a varied assortment of teaching contexts. They held teaching positions across different educational tiers, covering primary, secondary, and higher education levels.

The cohort encompassed 114 male participants (25%) and 342 female participants (75%). These teachers varied widely in age, with the youngest participant being 22 years old and the oldest 58, with a mean age of 34.82 years. They also exhibited a wide range of teaching experience, with a minimum of 1 year and a maximum of 47 years, and an average teaching experience of 10.91 years.

In terms of their academic qualifications, 250 individuals (54.82%) held Bachelor’s degrees, demonstrating a strong foundational education in their field. Furthermore, 190 participants (41.67%) had pursued and earned Master’s degrees, showcasing a commitment to further specialization in English language teaching. A smaller yet significant number of teachers, eight (1.75%), had attained the highest level of academic achievement with Ph.D. degrees, signifying a deep expertise in their area of study. An additional 8 participants (1.75%) boasted other educational attainments, potentially including teaching certifications and postgraduate diplomas, further enriching the diversity of the cohort.

These participants had diverse areas of expertise, with 218 specializing in Linguistics, 36 in Applied Linguistics, 20 in Translation Studies, 12 in TESOL (Teaching English to Speakers of Other Languages), 72 in TEFL (Teaching English as a Foreign Language), and 98 in other areas related to English language education. This diversity in academic qualifications and majors within the cohort offers a rich tapestry of perspectives and experiences, contributing to the depth and breadth of the study’s findings.

To ensure the adequacy of the sample size, a combination of statistical and practical considerations was employed. A power analysis was conducted using G*Power 3.1.9.2 (Faul et al., 2007) to determine the minimum sample size required to detect significant relationships between gratitude, job crafting, teacher-student relationships, and teacher psychological well-being. Based on the assumed medium effect size (f^2^ = 0.15) and a power of 0.80, the power analysis indicated that a sample size of 456 participants would be sufficient to yield reliable and generalizable findings.

Approaching and engaging EFL teachers for this study involved a comprehensive strategy aimed at ensuring their voluntary and informed participation. The study spanned a semester, approximately 3.5 months, with data collection condensed into a focused one-week period. Utilizing the established online platform ‘Questionnaire Star’ within China facilitated the administration of the questionnaire. To accommodate language preferences, both Chinese and English versions of the questionnaire were offered to ensure data accuracy and participant comfort. Rigorous validation of the translated scale was conducted through meticulous scrutiny by two translation experts to ascertain its linguistic and conceptual validity.

Prior to their involvement, detailed information regarding the research objectives was provided to the EFL teachers. Emphasis was placed on confidentiality, assuring participants that their responses and personal information would remain secure. Additionally, teachers were explicitly informed of their autonomy to withdraw from the study at any stage without requiring justification, ensuring voluntary and respectful participation.

### 3.2 Measures

#### 3.2.1 Job crafting scale

The examination of job crafting, a central component of our research, was conducted using the job crafting questionnaire established by Slemp and Vella-Brodrick (2013). Previous studies involving Chinese educators have documented favorable internal consistency results (Zhao and Li, 2019). The job crafting questionnaire encompasses three main dimensions: task crafting, relationship crafting, and cognitive crafting. This survey consists of 15 items, and participants were asked to rate each on a 5-point Likert scale, varying from 1 (Never) to 5 (Always). An example of a statement from this questionnaire is as follows: “I actively engage in the organization and participation of work-related social events.”

#### 3.2.2 Gratitude scale

Dispositional gratitude, a vital aspect of the study, was evaluated using the scale developed by McCullough et al. (2002). This questionnaire comprises six items rated on a 5-point Likert scale, ranging from 1 (Disagree) to 5 (Agree). Prior research with teachers has demonstrated the questionnaire’s good validity and reliability (Wei et al., 2011).

#### 3.2.3 Teacher-student relationship scale (TSRS-C)

In order to assess the quality of teacher-student relationships, we utilized the Chinese adaptation of the Teacher-Student Relationship Scale (TSRS-C; Chu, 2006). This instrument comprises 18 items, which are further categorized into three subscales: Situation, Intimacy, and Equality. Participants were asked to provide their ratings on a 5-point scale, ranging from 0 (“Strongly disagree”) to 4 (“Strongly agree”), with higher scores indicative of a more robust teacher-student relationship. An example item included in this scale is: “I frequently faced difficulty in comprehending the teacher’s explanations.”

#### 3.2.4 Psychological well-being scale

Psychological well-being was assessed using the psychological well-being scale developed by Ryff and Keyes (1995). This scale consists of 18 items that measure various aspects of well-being, including positive relationships with others, personal growth, self-acceptance, purpose in life, autonomy, and environmental mastery. The response options ranged from 1 (“Strongly disagreed”) to 7 (“Strongly agreed”).

### 3.3 Data analysis

To explore the relationships among gratitude, job crafting, teacher-student relationships, and teacher psychological well-being, a rigorous analytical approach was essential. Our choice of employing SPSS 28.0 for initial descriptive statistics and correlation calculations ensured a comprehensive understanding of variable relationships within the dataset.

To evaluate the construct validity of our theoretical model, Confirmatory Factor Analysis (CFA) using AMOS 26.0 was conducted. CFA was chosen for its ability to scrutinize the alignment between our hypothesized model and empirical data, ensuring the robustness of our measurement models. Further, Structural Equation Modeling (SEM) was employed to thoroughly explore the complex relationships between latent constructs. The decision to utilize SEM was rooted in its capability to simultaneously examine multiple relationships, offering a comprehensive understanding of the interplay between gratitude, job crafting, teacher-student relationships, and psychological well-being.

The model fit was assessed based on established metrics following Kline’s (2015) recommendations. Criteria such as a χ2/df ratio < 3 (*p* > 0.05), GFI, CFI ≥ 0.90, RMSEA <0.08, and SRMR <0.10 were utilized to ascertain the adequacy of model fits (Hu and Bentler, 1999), ensuring the reliability and validity of the proposed structural relationships.

## 4 Results

Table 1 provides descriptive statistics and correlations for the study variables. Job crafting has a mean score of 3.39 (SD = 0.80, *α* = 0.89), while gratitude has a mean of 2.97 (SD = 0.59, *α* = 0.81), teacher-student relationship has a mean of 3.72 (SD = 0.81, *α* = 0.90), and well-being has a mean of 3.68 (SD = 0.69, *α* = 0.94). In terms of correlations, job crafting significantly correlates with gratitude (*r* = 0.291, *p* < 0.05), the teacher-student relationship (*r* = 0.452, *p* < 0.01), and well-being (*r* = 0.348, *p* < 0.01). Gratitude is positively correlated with the teacher-student relationship (*r* = 0.546, *p* < 0.01) and well-being (*r* = 0.253, *p* < 0.05). Additionally, the teacher-student relationship is significantly correlated with well-being (*r* = 0.516, *p* < 0.01). The reliability coefficients also indicated that all scales had high internal consistency.

**Table 1:** Descriptive analysis and correlations.

	Mean	*SD*	Croanbach’s α	1	2	3	4
1. Job crafting	3.39	0.80	0.89	1			
2. Gratitude	2.97	0.59	0.81	0.291*	1		
3. T-S relationship	3.72	0.81	0.90	0.452**	0.546**	1	
4. Well-being	3.68	0.69	0.94	0.348**	0.253*	0.516**	1

T-S relationshi*p* = teacher-student relationship **p < 0.01, *p < 0.05.

Furthermore, we conducted confirmatory factor analyses to assess the appropriateness of the latent variables’ structure. During this examination, we contrasted the initially suggested one-factor measurement model with three alternative models.

The results, detailed in Table 2, unequivocally indicate that the initially proposed four-factor measurement model aligns more effectively with the observed data. Notably, the model yielded the following fit indices: *χ*^2^ = 684.329, df = 510, *p* < 0.001, CFI = 0.956, GFI = 0.906, RMSEA = 0.034, and SRM*R* = 0.189, signifying its superior fit.

**Table 2:** The measurement model analysis.

Models	*χ* ^2^	df	CFI	GFI	RMSEA	SRMR
Single-factor model (a)	738.561	512	0.935	0.879	0.042	0.225
Two-factor model (b)	799.122	517	0.921	0.854	0.046	0.262
Three-factor model (c)	726.874	514	0.942	0.888	0.039	0.205
Four-factor model (d)	684.329	510	0.956	0.906	0.034	0.189

a: All the constructs are regarded as single factor.

b: All constructs are regarded as one factor and job crafting regarded as separate factor.

c: T-S relationship and well-being are regarded as one factor and gratitude and job crafting regarded as separate factors.

d: All the constructs are regarded as separate factors.

The assessment of convergent and divergent validity was conducted according to the criteria established by Henseler et al. (2015), and the results are summarized in Table 3.

**Table 3:** Convergent and discriminant validity.

	AVE	MSW	ASW	CR
1. Job crafting	0.421	0.454	0.338	0.827
2. Gratitude	0.486	0.738	0.312	0.804
3. T-S relationship	0.347	0.377	0.290	0.832
4. Well-being	0.561	0.469	0.342	0.849

Convergent validity, which measures the extent to which different indicators of the same construct are related, is well-supported, as all Average Variance Extracted (AVE) values exceed the recommended threshold of 0.5. This suggests that a significant proportion of the variance in each construct is accounted for by its respective indicators. Specifically, job crafting demonstrates an AVE of 0.421, gratitude has an AVE of 0.486, T-S relationship exhibits an AVE of 0.347, and well-being displays the highest AVE of 0.561.

Furthermore, to establish discriminant validity, we examine the Maximum Shared Variance (MSV) and Average Shared Variance (ASV) values. In all cases, the MSV and ASV values are notably lower than their corresponding AVE values, confirming that the latent constructs are distinct from each other.

Lastly, the Composite Reliability (CR) values, which reflect the internal consistency reliability of the constructs, all surpass the acceptable threshold of 0.7. This indicates strong reliability for the measurement of each construct. Job crafting has a CR of 0.827, gratitude has a CR of 0.804, teacher-student relationship exhibits a CR of 0.832, and well-being demonstrates the highest CR of 0.849.

Upon successful validation of the measurement model, we proceeded to explore different structural models to evaluate our hypotheses. Specifically, we pitted the hypothesized partial mediation model (Model C) against both a full mediation model (Model B) and a direct model (Model A). The fit indices for all three models have been detailed in Table 4. The outcomes clearly indicated that the envisaged model (Model C) displayed a notably superior fit in comparison to Model B and Model A, as confirmed by the employed fit indices. Consequently, Model C was identified as the most optimal and concise representation for the data at hand.

**Table 4:** Fit indices of alternative models.

Model	*χ* ^2^	df	Δ*χ*^2^	GFI	CFI	RMSEA	TLI	SRMR
Direct effect model (A)	1265.789**	640	–	0.845	0.918	0.072	0.903	0.198
Full mediation model (B)	1468.876**	645	203.087	0.889	0.967	0.052	0.943	0.081
Partial mediation model (C)	1132.679**	641	227.211	0.906	0.982	0.041	0.960	0.068

Δ χ^2^ indicates the model discrepancies; **p < 0.001.

After establishing the validity of the measurement model, our focus shifted toward evaluating various structural models to rigorously examine our research hypotheses. Specifically, we compared three different models: the hypothesized partial mediation model (Model C), a full mediation model (Model B), and a direct model (Model A). A detailed summary of the fit indices for all three models can be found in Table 4.

The findings were unequivocal: the envisioned model (Model C) displayed a significantly superior fit when compared to both Model B and Model A, as substantiated by the employed fit indices. Consequently, Model C emerged as the most suitable and concise representation for the dataset we had gathered.

**Figure 1:**
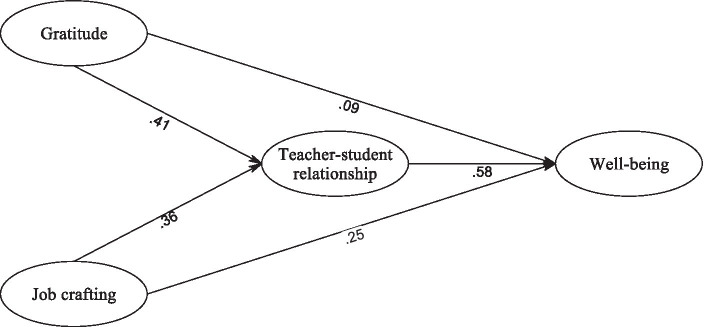
The fit model.

Figure 1 depicts the path diagram and parameter estimates for the final structural model (Model C), which exhibited a favorable fit to our data. In Figure 1, we can observe that all path coefficients were statistically significant, with one exception: the path connecting gratitude and well-being did not reach statistical significance.

The results of the structural model unveiled several significant findings. Firstly, there was a substantial positive relationship between job crafting and the teacher-student relationship (*β* = 0.361, *p* < 0.001). Similarly, gratitude displayed a significant association with the teacher-student relationship (*β* = 0.412, *p* < 0.001). Moreover, the analysis highlighted a positive link between the teacher-student relationship and well-being (*β* = 0.587, *p* < 0.001).

Subsequently, we explored the possibility of mediation by investigating the role of the teacher-student relationship between the various constructs, following the guidelines established by Baron and Kenny (1986). In the direct model (see Table 5), we identified substantial coefficients linking job crafting, gratitude, and well-being. These included the paths from job crafting to well-being (*β* = 0.421, *p* < 0.001) and from gratitude to well-being (*β* = 0.246, *p* < 0.05), thereby satisfying the first step of Baron and Kenny’s method.

**Table 5:** Model path analysis.

Normalized path weights (*t*-value)			
	Direct effects model	Full mediation model	Partial mediation model
Gratitude → well-being	0.246 (3.12*)		0.094 (1.21)
Job crafting → well-being	0.421 (4.57**)		0.258 (2.97**)
Gratitude → T-S relation		0.448 (5.14***)	0.412 (4.89***)
Job crafting → T-S relation		0.375 (4.09***)	0.361 (4.10***)
T-S relation → well-being		0.645 (7.18***)	0.587 (6.62***)

T-S relationshi*p* = teacher-student relationship; *p < 0.05, **p < 0.01, ***p < 0.001.

Additionally, our examination extended to the full mediation model, which revealed significant coefficients indicating the relationships between job crafting and gratitude with the teacher-student relationship. These coefficients were job crafting to the teacher-student relationship (*β* = 0.375, *p* < 0.001) and gratitude to the teacher-student relationship (*β* = 0.448, *p* < 0.001), affirming the second step of Baron and Kenny’s method.

Finally, the partial mediation model elucidated that job crafting exerted a positive influence on well-being (*β* = 0.258, *p* < 0.01). When considering the indirect effect mediated by the teacher-student relationship, we found that the impact of job crafting on well-being through the teacher-student relationship (0.361 × 0.587, i.e., 0.211) was smaller than the direct effect (0.211 < 0.258) of job crafting on well-being. This implies that the teacher-student relationship partially mediates the relationship between job crafting and well-being. In contrast, the path coefficient of gratitude on well-being was not statistically significant. However, the teacher-student relationship emerged as a full mediator between gratitude and well-being (0.412 × 0.587 = 0.241, which is greater than 0.094). This suggests that the impact of gratitude on well-being is channeled through the teacher-student relationship, positively influencing well-being.

To mitigate potential concerns of common method bias, we conducted Harman’s single-factor test by combining all latent variables measured through self-reported measures, including job crafting, gratitude, well-being, and the teacher-student relationship. The first factor extracted explained 42.03% of the variance, which falls below the 50% threshold. This outcome indicates that common method bias was not a significant issue in our study.

## 5 Discussion

In the pursuit of promoting teacher well-being, our study delves into the complex web of relationships between gratitude, job crafting, and teacher psychological well-being among Chinese EFL educators. The well-being of teachers holds profound significance not only for their individual quality of life but also for the overall effectiveness of the educational process. Against this backdrop, this study sets out to investigate the direct associations between gratitude, job crafting, and teacher psychological well-being, while also examining the mediating role of teacher-student relationships in these dynamics.

In this study, we found compelling evidence supporting the direct relationship between job crafting and teacher well-being. These findings are congruent with existing research in organizational psychology and education, which consistently emphasizes the positive impact of job crafting on employee well-being (Tims et al., 2012, 2022; Van Wingerden et al., 2017; Wang et al., 2022). Job crafting, as a proactive process allowing employees to tailor their roles to better suit their preferences and strengths, empowers individuals to exert control over their work environment, echoing the principles of Wrzesniewski and Dutton (2001) seminal work. In the context of teaching, this translates into teachers actively adjusting their responsibilities, customizing lesson plans, pursuing professional development opportunities, or initiating collaborative projects with colleagues.

The theoretical foundation for this effect is anchored in the Job Demands-Resources (JD-R) model, which posits that individuals adjust their work experiences to maximize resources while minimizing demands (Bakker and Demerouti, 2007). For teachers, this process heightens their sense of autonomy and control over their professional roles, ultimately leading to greater job satisfaction and enhanced well-being (Peral and Geldenhuys, 2016; Alonso et al., 2019). Our findings affirm that teachers who engage in job crafting report higher levels of well-being, underscoring the significance of this proactive approach in the context of EFL educators (Peral and Geldenhuys, 2016; Van Wingerden et al., 2017).

Moreover, this study contributes to the broader conversation about teacher well-being, a critical area of research and policy focus in education. Teacher well-being has far-reaching implications, impacting not only the job satisfaction and retention of educators but also the quality of education delivered to students (Skaalvik and Skaalvik, 2017; Greenier et al., 2021). By recognizing the role of job crafting in enhancing teacher well-being, educational institutions and policymakers can implement strategies and initiatives that promote a work environment conducive to job crafting, thereby fostering well-being among teachers (Peral and Geldenhuys, 2016).

In addition, our study provides robust support for the direct association between gratitude and teacher well-being. These results align with a growing body of research in the fields of positive psychology, education, and organizational psychology, which underscores the positive influence of gratitude on well-being (Chan, 2013; Howells, 2014; Komase et al., 2021). In the challenging context of teaching, where educators regularly confront various stressors and obstacles, the role of gratitude as a psychological resource for enhancing well-being is particularly pertinent.

Gratitude, a multifaceted construct involving the acknowledgment and appreciation of life’s positive aspects and experiences, is a potent tool for teachers (Wood et al., 2010; Komase et al., 2021). In the context of teaching, gratitude can manifest in various forms, including appreciation toward students, colleagues, and the opportunity to make a difference in students’ lives. Cultivating gratitude empowers teachers to shift their focus away from the inherent stressors and challenges of the profession, instead directing their attention toward the positive aspects of their work. This shift in perspective contributes to increased job satisfaction, reduced burnout, and ultimately, enhanced well-being (Chan, 2010a,b; Howells, 2014).

The literature offers substantial support for gratitude as a powerful positive emotion that can be harnessed to enhance well-being. Gratitude interventions, such as gratitude journaling, have consistently been linked to increased life satisfaction, happiness, and overall well-being (Rash et al., 2011; Chan, 2013). In educational contexts, research demonstrates that teachers actively practicing gratitude report greater job satisfaction, improved emotional well-being, and a more optimistic outlook regarding their teaching careers (Fagley and Adler, 2012; Bono et al., 2013).

Furthermore, as confirmed by SEM results, this study reveals a substantial and noteworthy mediation effect of the teacher-student relationship in the hypothesized model. Teacher-student relationships have long been acknowledged as a critical factor in the educational context, encompassing the emotional and interpersonal bonds between teachers and their students (Pianta et al., 2012; Lin et al., 2022). These relationships significantly influence the teaching and learning processes (Roorda et al., 2011; Fabris et al., 2023). The mediating effect of the teacher-student relationship observed in our study aligns with previous research, indicating that the quality of these relationships plays a pivotal role in teacher well-being (Farhah et al., 2021; Falk et al., 2022; Zheng, 2022).

According to JD-R model, job resources, such as social support and positive relationships, can act as buffers against job demands and contribute to employee well-being (Bakker and Demerouti, 2007). In the teaching context, job crafting represents a resource as it enables teachers to modify their roles and responsibilities to align with their preferences and strengths (Tims et al., 2012; Alonso et al., 2019). Job crafting empowers teachers to create a more positive and supportive classroom environment, which, in turn, leads to enhanced teacher-student relationships (Lazazzara et al., 2020; Dreer, 2022).

A strong teacher-student relationship can contribute to teacher well-being by fostering a positive and engaging classroom atmosphere, reducing teacher stress, and enhancing job satisfaction (Roorda et al., 2011; Farhah et al., 2021). When teachers have the autonomy to customize their teaching practices through job crafting, they are better equipped to tailor their interactions with students to meet their needs. This, in turn, leads to stronger teacher-student relationships and ultimately increases teacher well-being (Harju et al., 2021; Dreer, 2022).

Finally, the findings of this study illuminate a crucial mediation effect of the teacher-student relationship on the relationship between gratitude and teacher well-being. This result aligns with established literature on the influential role of teacher-student relationships in the well-being of both teachers and students, highlighting the significance of gratitude in enhancing these relationships (Aldrup et al., 2018; Zheng, 2022). Teacher-student relationships are widely recognized as a pivotal factor in the educational context, encompassing emotional and interpersonal bonds that play a vital role in the teaching and learning processes (Roorda et al., 2011; Longobardi et al., 2021). The mediating effect of the teacher-student relationship found in our study resonates with prior research, indicating that the quality of these relationships significantly affects teacher well-being (Aldrup et al., 2018; Farhah et al., 2021; Falk et al., 2022).

Gratitude, as a positive emotion, involves recognizing and appreciating the positive aspects of one’s life and experiences (Wood et al., 2010; Rash et al., 2011; Sun and Kong, 2013). In the context of teaching, gratitude can manifest in various forms, including gratitude toward students for their efforts and contributions to the learning process (Chan, 2010b). Grateful teachers may be more empathetic, compassionate, and supportive, fostering a positive and engaging classroom atmosphere (Chan, 2010a,b; Howells, 2014; Komase et al., 2021). Consequently, this contributes to the development of strong teacher-student relationships. Strong teacher-student relationships offer numerous benefits to teachers, such as reduced burnout, increased job satisfaction, and greater overall well-being. Additionally, these relationships contribute to a positive and engaging classroom environment, which facilitates effective teaching and learning (Roorda et al., 2011; Pianta et al., 2012). The mediating role of the teacher-student relationship between gratitude and teacher well-being is significant, suggesting that nurturing positive relationships with students enhances teacher well-being and, in turn, positively affects educational outcomes (Portocarrero et al., 2020).

## 6 Conclusion and implications

In conclusion, this study has contributed to a deeper understanding of the factors that impact teacher well-being, shedding light on the direct associations between job crafting, gratitude, and teacher well-being. Additionally, the mediation effects of the teacher-student relationship have been established, emphasizing the critical role of interpersonal connections in the teaching profession.

The direct relationships between job crafting and gratitude with teacher well-being highlight the potential for interventions and strategies to enhance teacher well-being. Job crafting empowers teachers to tailor their roles, fostering autonomy and control over their professional lives. Gratitude, as a positive emotion, encourages a shift in perspective toward the positive aspects of teaching, ultimately reducing stress and enhancing well-being. Recognizing the significance of these factors can inform policies and initiatives aimed at improving the well-being of educators, ultimately benefiting both teachers and their students.

The implications stemming from this study hold significant practical and theoretical relevance, advocating for actionable steps and theoretical insights to enhance the well-being of educators and foster a conducive teaching environment. Practically, educational institutions and policymakers can leverage the study’s findings as a call to action, recognizing the pivotal impact of job crafting and gratitude on teacher well-being. To facilitate this, interventions tailored to promote job crafting and gratitude practices among teachers can be instrumental. Professional development programs aimed at empowering educators to customize their roles and responsibilities could be devised. These initiatives might encompass flexible lesson planning, collaborative project opportunities, and autonomy in selecting teaching methodologies. By affording teachers the agency to shape their professional lives, educational institutions can significantly contribute to bolstering teacher well-being and job satisfaction.

Moreover, strategic initiatives promoting gratitude practices among educators hold promise. Integrating gratitude journaling, mindfulness training, or workshops centered around positive psychology into teacher training and professional development programs could be valuable. Encouraging teachers to embrace a gratitude mindset can foster a more positive and supportive teaching environment. This shift in perspective could lead to increased job satisfaction, reduced stress levels, and enhanced overall well-being for educators. Importantly, a positive teaching environment created through these practices can profoundly impact student experiences and learning outcomes.

Theoretical implications underscore the significance of integrating positive psychological concepts like gratitude and job crafting into the realm of education. By emphasizing the role of these factors in teacher well-being, this study contributes to the burgeoning field of positive organizational behavior within educational contexts. It provides a theoretical foundation for understanding how fostering gratitude and enabling job crafting practices can positively influence teacher well-being, highlighting their potential as valuable constructs for further research and exploration in educational psychology and organizational behavior studies.

Although this study provides valuable insights, several limitations should be acknowledged. First, the study design was cross-sectional, which means it can establish associations but not causality. Future research using longitudinal or experimental designs would be beneficial in establishing causal relationships between the variables studied. Second, the study relied on self-report measures, which may introduce response bias and common method variance. The use of objective or observational measures could provide more robust data. Additionally, the generalizability of the findings may be limited to the specific context of this study. Variations in educational systems, cultures, and teaching environments may influence the relationships studied. Future research should consider diverse contexts to ensure the applicability of the findings. Finally, other unmeasured variables may influence the relationships under investigation. Factors such as school culture, administrative support, and external stressors may impact teacher well-being and should be considered in future research.

## Data availability statement

The original contributions presented in the study are included in the article/supplementary material, further inquiries can be directed to the corresponding author.

## Ethics statement

The studies involving humans were approved by the Public English Teaching and Research Department, Qiqihar University, Qiqihar 016000, China. The studies were conducted in accordance with the local legislation and institutional requirements. The participants provided their written informed consent to participate in this study.

## Author contributions

XZ: Conceptualization, Data curation, Formal analysis, Investigation, Methodology, Project administration, Resources, Software, Supervision, Validation, Visualization, Writing – original draft, Writing – review & editing. HH: Conceptualization, Data curation, Formal analysis, Investigation, Methodology, Project administration, Resources, Visualization, Writing – original draft, Writing – review & editing. QY: Data curation, Investigation, Methodology, Project administration, Resources, Software, Supervision, Validation, Visualization, Writing – original draft, Writing – review & editing.
